# A Mentalization-Informed Staging Approach to Clinical High Risk for Psychosis

**DOI:** 10.3389/fpsyt.2019.00385

**Published:** 2019-05-31

**Authors:** Marco Armando, Joost Hutsebaut, Martin Debbané

**Affiliations:** ^1^Department of Psychiatry, Developmental Imaging and Psychopathology Lab, University of Geneva, Geneva, Switzerland; ^2^Viersprong Institute for Studies on Personality Disorders (VISPD), MBT Netherlands, Amsterdam, Netherlands; ^3^Department of Clinical, Educational and Health Psychology, University College London, London, United Kingdom; ^4^Developmental Clinical Psychology Unit, Faculty of Psychology, University of Geneva, Geneva, Switzerland

**Keywords:** schizophrenia, treatment, early intervention, mentalizing, social functioning

## Abstract

The practice of diagnosis is fundamentally designed to orient treatment. In the case of early diagnosis for schizophrenia spectrum disorders (SSP) risk, the empirical base for such a practice is still young, and many clinical questions arise in the everyday clinical application of risk algorithms and ensuing therapeutic options. One of the key questions that we will focus on is the following: in cases of SSP where symptoms are successfully treated, why does residual social functioning impairment remain the most serious obstacle to remission and reinsertion in society? We will present the evidence suggesting that the roots of residual social functioning impairment may, in many cases, come from thwarted or arrested development in the specialization of social cognition during adolescence and early adulthood. We will review the evidence suggesting that both during the premorbid phase and clinical high-risk phase, attenuated psychotic symptoms may impede the maturation of key social cognitive processes, particularly the suite of reflective thinking processes coming under the term of mentalization. From this evidence base, we will adapt the staging model of SSP progression in function of our mentalization-informed model, tailored to provide a coherent framework of care addressing the key clinical needs at every stage of psychosis progression.

## Background

In its short history, the topic of early diagnosis for clinical high-risk states to develop psychosis (CHR-P) has stirred both hope and controversy. Early diagnosis aiming to shorten the duration of untreated psychosis (DUP) proved valuable to improve outcome along the clinical course of schizophrenia spectrum disorders (SSP) (1). Powered by research on DUP, attempts to characterize the psychological risk states preceding psychosis were received with both enthusiasm and opposition. While an early diagnosis of prodromal states could justify indicated preventive treatment, intense debate and vigorous opposition appeared fuelled by concerns for the validity of the risk constructs, fear for diagnosing false positives and for the effects of labelling (2). Today, expert consensus puts forward validated tools to clinically assess CHR-P states preceding the onset of SSP (3); the clinical practice of these tools and the initiation of early treatment is currently exerted with caution (4). In this article, we wish to reframe the questions surrounding early diagnosis and early treatment to the following question: which type of clinical care is needed at different stages of the progression of psychosis? A central empirical finding will guide our discussion on this question: regardless of symptomatic remission in SSP, residual social functioning impairment remains the most serious obstacle to full recovery and reinsertion in society. We will present the evidence suggesting that the roots of residual social functioning impairment may, in many cases, come from thwarted or arrested development in the specialization of social cognition during adolescence and early adulthood. We will review the evidence that many factors along the preclinical phase of psychosis may impede the maturation of key social cognitive processes, particularly the suite of reflective thinking processes coming under the term of mentalization. From this evidence base, we will revise the staging model of psychosis progression, and outline our mentalization-informed approached tailored to provide a coherent framework of care addressing the key clinical needs at every stage of psychosis progression.

## Why Early Diagnosis?

In the past quarter of a century, research on the risk of developing schizophrenia spectrum and other SSPs [Diagnostic and Statistical Manual of Mental Disorders (DSM-5)] has dramatically transformed our clinical and scientific approach to what Eugene Bleuler referred to as the « schizophrenias » (5). In concert with emerging findings in developmental neuroscience, these disorders, which we regroup under the rubric of SSP, are now conceptualized as neurodevelopmental in origin (6). Importantly, expert agreement situates the development of SSP along four distinct periods: the premorbid phase, the clinical high-risk states, the first episode of psychosis, and the trajectories following the first diagnosis (7). The asymptomatic premorbid period during childhood and adolescence can be characterized by non-specific impairments in cognition (8), infra-clinical manifestations of trait risk such as negative and positive schizotypy or subtle cognitive disorganization (9–11), as well as slight social cognitive impairments (12, 13). The pathogenesis can evolve from the premorbid phase to a subclinical stage of risk symptoms preceding the actual onset of the disorder. These risk symptoms represent CHR-P states, which are reliably diagnosed through the use of validated instruments such as the CAARMS (Comprehensive Assessment of At-Risk Mental-States) (14), the SIPS (Structured Interview for Prodromal States) (15), and the SPI-CY/SPI-A for basic symptoms (Schizophrenia Proneness Instrument—Child and Youth version or Adult version) (16). Questions of diagnosis and early treatment are most controversial during this phase (17), when rates of CHR-P individuals not developing SSP can be high (18), diagnostic procedures focusing on different types of manifestations (19), notwithstanding that clinically speaking, the recognition of the psychotic nature of the risk may be difficult to perceive for the young person and her/his family.

Once a diagnosis of psychotic disorder is established, the treatment guidelines are clear (20), and potential issues with these guidelines lie beyond the scope of this article. Hence, the most debated issue of early diagnosis and treatment lies within the CHR-P period, and as we will suggest, can be extended to questions surrounding early prevention strategies in the premorbid phase. The diagnosis of CHR-P states, while widely practised and accepted by experts in the field, still fosters debate (21). While a categorical diagnosis of Attenuated Psychosis Syndrome (which correspond broadly to the CHR-P state) has been recently added in the section 3 of the DSM-5 (22), concerns for over-diagnosis are currently being researched (23), and conceptual debates confronting categorical versus continuum views of psychosis remain vigorous (2, 24).

The question of early treatment is closely linked to that of early diagnosis for a simple reason: ideally, a diagnosis should indicate clear treatment rationale and options. Yet in the present state of scientific advancement, research for treatment in CHR is only nascent (25). Additional issues originate from the point of view of public health: in many countries, health care systems will allocate resources to patients on the condition of a recognized medical diagnosis. In such instances, economic and political forces can both push for and/or pull away from the recognition of a condition (26). As it stands before the turn of the decade in 2020, the diagnosis of CHR (DSM-5) is still under observation, and as we have briefly summarized, a number of issues residing outside of the purely diagnostic debate still remain. While the question of early diagnosis is still open, an increasing number of studies point to two complementary pieces of evidence: 1) individuals diagnosed with a CHR-P state, but that do not transition to psychosis, still require clinical attention (27) and have worst outcome compared to those who didn’t experienced a CHR-P (28); 2) longitudinal research does not overwhelmingly support the notion of *transition to psychosis* as a key predictor of functional outcome (29). This poses the questions of the clinical needs of individuals with sub-threshold psychotic symptoms and comorbid disorders, and furthermore, which kind of treatment would be adapted to their clinical profiles.

## The Rationale for Early Treatment

One of the issues reaching beyond diagnosis relates to the question of whether treatments can be offered to people before the onset of psychosis, and if so, what should be the main measure of outcome to judge their efficacy?

A recent meta-analysis of psychological and psychopharmacological randomized-control trials (RCTs) for individuals meeting the established criteria for CHR-P states a clear and structured perspective on the studies performed over the past 10 years (4), also pointing to areas of potential amelioration in both research and clinical work with CHR-P. The meta-analysis focuses on the conversion rate to psychotic disorder as the principal outcome of their analysis, and further considers functional improvements as a key outcome to studies with these populations. Schmidt et al. find evidence that early intervention provides significant benefits to individuals at CHR-P in terms of either significantly preventing or delaying the emergence of a psychotic disorder. This result has been supported by more recent meta-analyses on early treatment with CHR-P (30).

Interestingly, however, the available meta-analyses examining early treatment during CHR-P find no treatment superiority effects when comparing psychological vs neuroleptic medication, nor any superiority effect within the different psychological treatments under study (4, 25, 30). The variety of types of treatment, at this stage, is moderate. In addition to psychopharmacological trials, RCTs have been performed for nutritional supplements, cognitive behavioural treatment, multi-family psychoeducation, and combined interventions with additional social skills training. Despite the important differences in methodology, no superiority effect was found for treatment type; this is consistent with recent reports on the “Dodo bird effect” in psychotherapy, using a variety of therapy models, for a variety of different psychiatric conditions (31). Perhaps most intriguing is the lack of superiority to control conditions with regards to functional improvements. Indeed, while specialized early treatment methods significantly decrease the transition rate to SSP, functional outcomes are roughly equivalent to those obtained through control conditions. It appears, therefore, that room for improvement in early treatment for psychosis is most apparent in the area of functional outcomes.

## Concern for Functional Outcomes and the Relevance of a Developmental Model

In many different domains of mental health treatment, a discrepancy can be observed between, on the one hand, an individual’s symptomatic improvement with treatment, and on the other hand, the same individual’s stability or worsening of adaptive functioning. In SSP, the functional outcome or global functioning beyond symptom severity represents the sum of several different but correlated domains, such as cognitive, role and social functioning. Further, several studies suggest that impairments in social functioning create the most disability in SSP. Conversely, the “symptom-disability gap” observed over the course of treatment is often portrayed in the treatment of SSP (32). This gap is not unique to SSP, it can also be observed in other conditions, for example attention deficit hyperactivity disorder (ADHD) (33). The symptom-disability gap creates a clinical puzzle: how can interventions that attenuate symptoms fail to produce positive cross-over effects into functional improvements? Treatment for individuals with the full diagnosis of schizophrenia have provided the clearest examples of the symptom-disability gap: indeed, it is estimated that social functional impairments characterize more than half of patients with treatment (34). While symptoms appear to respond to medication, patients often fail to benefit from improvements in their daily-living conditions.

In SSP, the issue of intact impairment of social functioning regardless of treatment also seems to apply to treatment for earlier stages of the disease, notably treatment for youth at CHR. In a recent meta-analysis of early interventions for youths at CHR for psychosis, Devoe et al. specifically examine the effect of preventive treatment on social functioning (35). The meta-analysis included 19 trials encompassing 1,513 patients meeting diagnostic criteria for CHR-P. Neither cognitive behavioural trials, nor omega-3 trials and cognitive remediation trials significantly improved social functioning in youth at CHR-P. The authors remark on the need to adapt early interventions to the domains of functioning, namely social functioning, that require support in the early stages of psychopathological progression to psychosis.

As we have argued elsewhere (36, 37), targeting social cognition in early interventions constitutes a challenging ambition, especially in the case of youths at CHR-P. Firstly, decades of research on socio-emotional development during adolescence and early adulthood suggest that a number of different processes interact to promote growth and socially adaptive behaviour. In parallel, cerebral maturation during the same age period will sculpt the morphological brain areas, contribute to the specialization of skills needed to function at high levels of social complexity, and fuel the integration of complex neuro-functional networks that will sustain continued maturation of social functioning skills (38, 39). Critically, within this same period of adolescence and early adulthood, youths in the premorbid stage can already show signs of subtle impairments on a range of skills sustaining social cognition, that is, the set of skills that enable to perceive, analyse, interpret and select adaptive behaviours in interpersonal and social contexts (40, 41). This set of skills can be subsumed under the construct of *mentalization*, that is, the suite of social cognitive imaginative activity enabling the interpretation of behaviour in terms of intentional mental states (42). Mentalization confers the possibility of imagining the intentions, emotions, motivations, and beliefs behind others’ actions, as well as behaviours of oneself that are more complex to understand or justify. It is crucial for social understanding and adaptation and, in evolutionary terms, it is thought to have evolved out of the need for human collaboration and competition (43). Thinking about mental states underlying individual actions can provide the necessary tools for anticipating behaviour, understanding relationship patterns, and adapting to different types of social environments (44). Recent neuroscientific research has shown how adolescence constitutes a key developmental window for the integration of the neuro-functional networks that articulate the processes to sustain accurate mentalizing (44–46).

In a similar line of thought, but focusing on the origin of social cognitive impairments in clinical samples, current research points out that the relationship between impaired social cognition and psychosis does not originate from secondary deficits associated with chronic psychosis, nor does it constitute a consequence of first episode psychosis, because impairments in social cognition are already apparent during the stage of CHR-P (47), and can be found to be predictive of conversion to psychosis, notwithstanding that more subtle impairments have been associated to the premorbid phase (48–50). In parallel, several reports have suggested subtle early impairments in a number of different social cognitive processes contributing to mentalizing. From the point of view of neurodevelopment, aberrant maturation of the right superior frontal, middle frontal, and medial orbitofrontal predict conversion to psychosis in CHR-P (51); these regions are best known to sustain mentalizing (52). Behavioural evidence of developing mentalizing skills in youths suggest, first, that in 11–12 year olds who report auditory verbal associations (a symptom of positive schizotypy), present faulty inferences of others’ mental states in the form of hypermentalizing (12, 13), that is, providing mentalistic assumptions clearly beyond the available evidence. Second, a number of studies reports impaired mentalizing in youths showing either trait risk, such as high schizotypy scores (53, 54), or state risk, such as CHR-P (55, 56). In a recent study on 632 CHR-P participants aged 12–35, evidence for reduced theory of mind (mentalizing the mind of others) could be evidenced as of 17 years of age (57), suggesting that adolescents and young adults with CHR-P can experience significant difficulties in understanding phenomena such as sarcasm and lies, which require specialized mentalizing skills that mature during adolescent development.

The nature of the relationship between impairments in social cognition and manifestations of psychosis from the premorbid to the clinical stages of expression still remains unclear. If the developmental process of social cognition is independent from the pathogenesis process of psychosis, then impaired social cognition may simply reflect the impact of pathogenesis at every stage in association to neurodevelopment (49). If impaired social cognition interacts with pathogenesis, as hypothesized by several authors including Paul H. Meehl’s theory of schizotypy (58, 59), then the best explanatory model would be one of the interacting processes leading to psychopathological outcome.

Another hypothesis, complementary to the first two, which stands on the idea of a synergy between psychotic symptoms and lack of social cognition, has recently been put forward by our group: we may conjecture that progressing psychotic pathogenesis impacts the very development of social cognitive processes, and vice versa (37). Indeed, the expression of negative schizotypy such as in physical and social anhedonia, social anxiety and social withdrawal may each impact the very opportunities of interpersonal and social interactions during adolescence and young adulthood. Anhedonia affects the motivational system responsible for triggering anticipated interest in interpersonal exchange, and impedes the allowance of cognitive resources to understand how minds work and how they influence behaviour. Social anxiety will affect the behavioural predisposition to seek out interpersonal and social exchange by fostering social avoidance. Finally, social withdrawal may affect the establishment and maintenance of close interpersonal relationships, a context in which significant interpersonal and social understanding can be experienced and deepened. Thus, many of the key manifestations in distal risk for psychosis (60) already affect the creation of the psychological tools to seek, participate in, and understand the interpersonal social world.

In the opposite but complementary direction, the development of mentalizing seems to confer a protective role in those individuals at risk of developing psychosis. In a longitudinal study among children experiencing auditory hallucinations at ages 7–8 and/or 12–13, Bartels-Velthuis et al. found that the development of delusional ideation secondary to abnormal perceptual experiences (AVH) was reduced when participants demonstrated strong mentalizing skills (61), hinting to the protective nature of strong mentalizing skills early in development. Furthermore, robust mentalizing may also reduce the distress caused by psychotic symptoms, as suggested recently by Peters et al. (62). In this original study comparing non-schizophrenic but persistent voice hearers to voice-hearers with schizophrenia and to non voice-hearing controls, the study investigated which kind of features might reliably distinguish between these three groups. While testing for a variety of clinical, socio-demographical and psychological characteristics, the study, which enrolled almost 100 participants in each group, found that the only psychological process distinguishing persistent but non-schizophrenic voice hearers from both controls and voice-hearers with schizophrenia was mindfulness, as measured by the Southampton Mindfulness Questionnaire (SMQ) (63). Indeed, the non-clinical voice-hearers reported higher mindful responding to internal thoughts and images in comparison to clinical voice-hearers, but what is more surprising is their increased mindfulness skills in comparison to controls. Mindfulness is directly linked to mentalizing one’s own thought content, and cultivating a relationship of curiosity and acceptance with the production of one’s mind (64). This study underlines that mentalizing others, as measured in ToM tasks, is not the only dimension of mentalizing that is key to resilience processes. Indeed, as we have suggested elsewhere, mentalizing oneself may be especially important in relation to risks for psychosis, because individuals on the clinical continuum of psychosis experience disturbing stimuli that is self-generated (self-criticism, paranoia, thought disorganisation, or disturbing sensory or perceptual experiences for example) that do not temporally respond to contingent and upsetting emotional stimulation by others, more typical in emotional arousal observed for borderline personality disorders (36, 37). As suggested by these reports and others [for a review, see Ref. (37)], both self and other mentalizing may thus constitute potent protective factors in the face of risk for psychosis. These different strands of evidence also underline the utility of the concept of mentalization, which unifies different psychological constructs related to thinking about mental states into a coherent framework articulated to a therapy model (65, 66).

Indeed among the therapeutic models adapted to focus on the early impairments in social cognition, Mentalization-Based Therapy (MBT) constitutes an integrative intervention first developed to address psychotherapeutic treatment for Borderline Personality Disorder (BPD), and more recently successfully adapted to psychological treatment for the most severe psychopathologies in adolescents and adults (67, 68). The model aims to increase the client’s capacity to mentalize, that is, to identify mental states in oneself and others, and reflectively assess their contributions to patterns of dysregulated behaviour, emotional reactions, or maladaptive thought patterns. Three main reasons would sustain the pertinence of such a model for psychosis along its different stages of clinical evolution. First, recent studies on MBT adapted for patients with non-affective SSPs have shown feasibility and promising results (69, 70). Furthermore, MBT has proven to be effective in adolescent conditions which typically present sub-clinical psychotic symptoms (68). As such, the same model of therapy is applicable to the range of clinical manifestations along the continuum of psychosis expression. Randomized controlled trials (RCTs) conducted with patients suffering from borderline personality disorder have shown its therapeutic effect on interpersonal relationships and emotion regulation processes (71–73). More recently, MBT has been successfully adapted to a range of disorders (74), and interestingly, an increasing number of reports relate successful attempts to adapt MBT for CHR-P (36, 37) and SSP (75–77). In line with this preliminary evidence, we next sketch out a mentalization-informed staging approach to psychosis, from the premorbid to the full diagnostic conditions.

## A Mentalization-Informed Staging Approach to Psychosis

Broadly speaking, the clinical staging model is a trans-diagnostic heuristic approach aimed at understanding the neurobiological and environmental processes underpinning the onset and course of a disorder. Clinical staging, through integrating stage and timing with evolution of clinical phenotype, also allows interventions to be tested from a preventive standpoint in reducing the risk of progression and persistence of illness. The idea of a clinical staging approach for psychosis as a progressively intensive intervention model aimed at prevent/delay the transition to psychosis in CHR-P subjects has been firstly developed by McGorry and colleagues more than 10 years ago (78). This model was originally focused on preventing the progression of psychotic symptomatology through different levels of intervention (starting from psychosocial interventions up to antipsychotic medications), in accordance with the severity of the symptomatology. Even if the most recent developments of the clinical staging model for psychosis (79) have broadened the outcomes of interest moving from symptoms to functioning, the proposed interventions are more focused on treating symptoms instead of intervening directly on psychological processes that sustain resilience, such as mentalization.

In accordance and as a consequence of the hypothesis that we formulated in the previous section, we propose a revised staging model, which is more focused on treating the progressive delay/impairment in social cognition (social functioning) alongside the monitoring of potentially progressing psychotic (and others) symptoms. As showed in **Table 1**, together with the clinical progression of the psychotic symptomatology from stage 0 (subtle, subjective, non clinical pre-psychotic experiences such as psychotic-like experiences (PLEs), anomalous self experiences (ASE), basic symptoms (BS), NSS) to stage III (chronic psychosis), we propose a model of progressive impairments mentalizing skills which filtrates in parallel (in synergy) to progressing symptomatology. This progression starts by slightly affecting interpersonal, academic and social functioning, may increase by perturbing the ability to interpret social interactions (further affecting interpersonal, academic, and social functioning), and can lead to an arrest in the development of mentalizing competences, with ensuing consequences much later in the outcome of trajectories with psychosis.

**Table 1:** Clinical staging model of psychosis with focus on progressive social disfunctions (deficits of social understanding).

Stage	Clinical description	Persistence	Pervasiveness	Social functioning
Stage 0	Psychotic like experiences, basic symptoms, anomalous self experiences, soft neurological signs, cognitive and negative symptoms.	Pre-morbid	Non specific problems with subtle impairments in social cognition	Affects school functioning and social integration with peers (physical and social anhedonia, reduced peer contact, social anxiety)
Stage Ia	Attenuated psychotic symptoms, negative, neurocognitive and social cognitive symptoms, depressed mood and other psychological and behavioural abnormalities.	Duration of attenuated symptoms is limited, ability to discriminate between ideas and perception, fantasy partially preserved	Possibly axis 1 clinical disorders, like mood or anxiety disorders	Affects school functioning more severely (difficulties concentrating, peer contact, social anxiety)
Stage Ib	Brief self-limiting psychotic symptoms, negative, neurocognitive and social cognitive symptoms, depressed mood and other psychological and behavioural abnormalities.	Duration of psychotic symptoms is limited, loss of ability to discriminate between ideas and perception, fantasy (during brief symptoms episodes)	Possibly axis 1 clinical disorders, like mood or anxiety disorders	Imminent developmental arrest (abscence from school, social withdrawal); more significant polarizations in mentalizing, affecting interpretation of social interatcions, problems arise at different life areas (school, peers, home)
Stage II	First episode of psychosis (FEP)	First episode of full-blown psychosis. Long term loss of ability to differentiate between reality and thoughts	Possibly axis 1 clinical disorders, like mood or anxiety disorders	Severe impact on social functioning and school functioning; severe arrest in development and severe impairments in mentalizing
Stage III	Chronic psychosis	Chronic duration of total illness, progressive decline in cognitive and social functioning	Co-morbidity as a rule	Severe and chronic impairment in social and professional functioning; no or limited recovery

In **Table 2**, we attempt to provide an overview of a coherent and progressive MBT intervention model (MBT CHR-P) aimed at sustaining the development and safeguarding against impairments in mentalizing abilities in patients putatively at-risk for psychosis, at each stage of the clinical progression (**Table 1**). Broadly speaking, this intervention model is based on the 5 principles of the clinical staging model for CHR-P. Firstly, a staged approach to treatment is offered, with low intensity and least specialized interventions used initially, and “stronger,” more intensive interventions, reserved for those who do not respond to the earlier stages of intervention. In line with this approach and with the original clinical staging model for CHR-P, medications are considered as a possible intervention only starting from stage Ib. Moreover, there is still a lack of evidence concerning the efficacy of medications such antipsychotics or other molecules (eg. Omega-3 fatty acids; NAC) to prevent/delay transition to psychosis (80). Secondly, this strategy should address the problem of the low transition rate to psychosis (2). Indeed, this strategy is primarily addressed at improving mentalizing abilities and consequently the clinical and social functioning needs instead of mainly avoiding transition to psychosis. Nevertheless, this latter remains one of the targets of the model. Thirdly, this model is based on the hypothesis that the efficacy of therapeutic interventions that strengthen resilience (such as MBT) is closely correlated to the timing of the intervention. In this sense, the developmental phase (i.e. age, psychological maturity) as well as the stage of the disorder must be taken into account to decide the type of intervention. Fourthly, the model seeks to enhance compliance by addressing the therapeutic objectives of the patient themselves in the treatment formulation, which do not necessarily include attenuated psychotic manifestations, but issue such as patient-reported sources of distress such as anxiety and social functioning (81). In line with the fourth principle, this staged approach addresses ethical concerns, namely the potential stigma, the “false positive” issue, and a perceived relative lack of predictive power, by adapting the intervention to key clinical targets at every stage.

**Table 2:** Possible mentalizing interventions according to the clinical stage.

Stage	Interventions	Timing/Setting	Targets/Goals
Stage 0	School and family-based prevention	‘mental’ or ‘emotional’ education in primary prevention large scale campaigns	Providing psychoeducation about mentalization, the linkage between arousal, anxiety, reduction of cognitive performance and mentalization.
Stage Ia	MBT-A; MBT-F psycho-education	Short intervention, including psycho-education, skills strenghtening in adolescent groups and/or family therapy	More focused psychoeducation about the linkage between arousal, loss of mentalization and the development of psychotic symptoms. Patients are told about the key aspects of MBT, including the meaning of mentalizing and its sensitivity to arousal
Stage Ib	As for 1a Medications targeted to treat comorbid disorders (eg. anxiety, depression) Take into account low doses of antipsychotics in accordance to severity of BLIPS	More intensive intervention including individual work, combined with intervention at multiple levels (school, family,…)	Starting from stage Ib the intervention is progressively focused on 1) the patient’s state of mind as central to the rehabilitation of the capacity for social understanding; 2) the emphasis on the role of affect in disruptions of the ability to mentalize; and 3) the importance given to understanding the links between the quality of mentalization and specific Interpersonal/ attachment contexts. Five problem areas are developed and routinely reviewed with the patient, including: commitment to treatment, psychiatric symptoms, social interaction/relationships, destructive behavior, and community functioning
Stage II	As for Ib MBT-G for psychosis Antipsychotics	Long and intensive intervention
Stage III	As for II AMBIT	Very long intervention with an explicit focus on case management and an outreaching approach

MBT-A, Mentalization-Based Therapy Adolescents; MBT-F, Mentalization-Based Therapy Family; MBT-G, Mentalization-Based Therapy for Groups; AMBIT, Adaptive Mentalization-Based Integrative Treatment. AMBIT is a manualised mentalization based approach aimed at working with hard to reach people at risk of a wide range of life adversities. It uses mentalization as an organising framework for integrating a range of specific techniques and practices derived from different evidence based modalities of intervention. Integration is principally achieved through a focus on delivery of multiple modalities through a single worker, and mentalization-based practices developed to enhance team and network functioning.

We further attempt to integrate notions of primary and indicated selective prevention within the MBT-informed care plan. Referring again to **Table 2**, interventions for stage 0, a stage characterized by less severe and less specific clinical phenotypes, are tailored on the primary prevention that aims the education sector (82). In this early phase, interventions are provided at school and family levels and are aimed at sustaining the development of mentalizing skills and fostering a mentalizing environment which has a number of transversal benefits, such as reducing bullying and violence in schools (83, 84), and therefore may be relevant to legislators that would be less sensitive to a psychosis-targeted primary prevention program. The focus at this stage is really to enrich the traditional pedagogical stance with some mentalizing knowledge, and certain current schools-based experiments focus on integrating a mentalizing perspective within the educational context (85).

Moving along in **Table 2**, Stage Ia and Ib provide a progressively more intensive and specific intervention based on the MBT-adolescents (MBT-A) and MBT-family (MBT-F) model, along a selective prevention principle (36). MBT-A program and MBT-F are manualized, psychodynamic psychotherapy programs with roots in attachment theory [for descriptions of the interventions, see Refs. (68, 86, 87)]. During stage Ia, this intervention will be mostly provided for short periods and in a group setting. It involves weekly individual MBT-A sessions and monthly mentalization-based family therapy. During stage Ib, the MBT-A program will become more intensive and mostly structured on individual setting. In stage II and III, progressively more specific MBT intervention for SSP, such as MBT-G [see Ref. (69)] for psychosis and Adaptive Mentalization Based Integrative Treatment (AMBIT) (67, 88), which guides multidisciplinary teams working with hard to reach clinical cases using case manager models of care.

Overall, the mentalization-based approach to clinical staging of psychosis provides a coherent theoretical and clinical framework. This framework affords two key advantages: it provides a clinical intervention that is suited for both the psychotic related symptoms, but also the other psychiatric comorbidity issue that ultimately influence the severity of clinical outcome. Second, it provides a framework for training professionals that is applicable to a range of professionals who are susceptible to intervene at different stages of the progression of psychosis, from educators to general practitioners, as well as case managers, psychiatric nurses, psychologists and psychiatrists. The MBT model is based on the past 20 years of empirical research in child, adolescent and adult clinical interventions (89, 90). With regard to early diagnosis and treatment, the mentalization-informed model of staging we present here is specifically designed to promote development and prevent impairments in the key social cognitive processes before the onset of psychosis, in order to respond to the clinical needs of individuals “at-risk,” and further to attempt to ameliorate the poor long-term outcome of social functioning should individuals evolve towards a diagnosed psychotic disorder. Nevertheless, at this stage, the proposed MBT intervention based on the clinical staging model still needs to be tested and evaluated in clinical settings. Indeed, while there are some preliminary reports on the efficacy of MBT in FEP and in SSD (69, 91), there is still a lack of evidence concerning the ability of this intervention to reduce the transition to psychosis and ameliorate social functioning in patient at CHR-P (36).

Consequently, the usefulness of MBT in these early stages should be tested empirically with pilot randomized single-blind superiority trials comparing the efficacy of the MBT model with TAU in CHR-P adolescent population on several targets. Indeed, such trials should test firstly the 1) acceptability and attrition rate of the MBT model. Secondly, the efficacy of MBT in improving 2) mentalization abilities and 3) social functioning in CHR-P patients indipendently to transition to psychosis should confirmed. Thirdly, the efficacy of MBT in reducing 3) severity of psychotic symptoms and 4) transition to psychosis rates should be investigated. As a fourth step, the presence of biological substrates of the effect of MBT such as stress hormones and brain function (f-MRI) should be investigated in order to confirm the validity and the specificity of the MBT model.

## Conclusion

In providing a mentalization-informed framework for the staging of CHR-P and transition to psychosis, we attempt to target a key problem in the treatment of SSP, namely, the symptom-disability gap in outcomes of treatment where individuals still suffer from poor social functioning. We argue that the roots of residual social functioning impairment may, in many cases, come from thwarted or arrested development in the specialization of social cognition during adolescence and early adulthood. Our approach is also pragmatic, and sensitive to the cases of “non-conversion” to psychosis, for which important clinical care is still needed. Much of the clinical practice in developmental psychopathology is performed under conditions of uncertainty as to the symptomatic evolution and clinical outcome of individuals seeking help. Further clinical research that integrate the principles of good practice in the respect of empirical evidence will further sculpt the tools and methods of early diagnosis and intervention, to provide the most adapted care plan sustaining the development of the individual while attempting to divert the negative impact of psychosis progression on the interpersonal and social functioning domains, which today represent the key obstacles to therapeutic success with psychosis.

## Author Contributions

All three authors contributed to writing the manuscript.

## Funding

M.D. from the Swiss National Science Foundation (100019_159440).

## Conflict of Interest Statement

The authors declare that the research was conducted in the absence of any commercial or financial relationships that could be construed as a potential conflict of interest.

## References

[1] Perkins DO Gu H Boteva K Lieberman JA Relationship between duration of untreated psychosis and outcome in first-episode schizophrenia: a critical review and meta-analysis. Am J Psychiatry (2005) 162(10):1785–804. 10.1176/appi.ajp.162.10.1785 16199825

[2] van Os J Goluksuz S A critique of the “ultra-high risk” and “transition” paradigm. World Psychiatry (2017) 16(2):200–6. 10.1002/wps.20423 PMC5428198 28498576

[3] Schultze-Lutter F Michel C Schmidt SJ Schimmelmann BG Maric NP Salokangas RK EPA guidance on the early detection of clinical high risk states of psychoses. Eur Psychiatry (2015) 30(3):405–16. 10.1016/j.eurpsy.2015.01.010 25735810

[4] Schmidt SJ Schultze-Lutter F Schimmelmann BG Maric NP Salokangas RK Riecher-Rossler A EPA guidance on the early intervention in clinical high risk states of psychoses. Eur Psychiatry (2015) 30(3):388–404. 10.1016/j.eurpsy.2015.01.013 25749390

[5] Bleuler E Dementia praecox or the group of schizophrenias (J. Zinkin trans.). New York: International Universities Press (1911/1950).

[6] Rapoport JL Addington AM Frangou S Psych MR The neurodevelopmental model of schizophrenia: update 2005. Mol Psychiatry (2005) 10:434–49. 10.1038/sj.mp.4001642 15700048

[7] Debbané M Schizotypy: a developmental perspective. In: Mason O Claridge G, editors. Schizotypy: New Dimensions. Abingdon-on-Thames, United Kingdom: Routledge (2015).

[8] Reichenberg A Caspi A Harrington H Houts R Keefe RS Murray RM Static and dynamic cognitive deficits in childhood preceding adult schizophrenia: a 30-year study. Am J Psychiatry (2010) 167(2):160–9. 10.1176/appi.ajp.2009.09040574 PMC3552325 20048021

[9] Debbané M Badoud D Balanzin D Eliez S Broadly defined risk mental states during adolescence: disorganization mediates positive schizotypal expression. Schizophr Res (2013) 147(1):153–6. 10.1016/j.schres.2013.03.012 23570898

[10] Fluckiger R Ruhrmann S Debbane M Michel C Hubl D Schimmelmann BG Psychosis-predictive value of self-reported schizotypy in a clinical high-risk sample. J Abnorm Psychol (2016) 125(7):923–32. 10.1037/abn0000192 27583768

[11] Kwapil TR Gross GM Silvia PJ Barrantes-Vidal N Prediction of psychopathology and functional impairment by positive and negative schizotypy in the Chapmans’ ten-year longitudinal study. J Abnorm Psychol (2013) 122(3):807–15. 10.1037/a0033759 24016018

[12] Clemmensen L van Os J Skovgaard AM Vaever M Blijd-Hoogewys EMA Bartels-Velthuis AA Hyper-theory-of-mind in children with psychotic experiences. PLos One (2014) 9(11):e113082. 10.1371/journal.pone.0113082 25397582 PMC4232592

[13] Clemmensen L van Os J Drukker M Munkholm A Rimvall MK Vaever M Psychotic experiences and hyper-theory-of-mind in preadolescence - a birth cohort study. Psychol Med (2016) 46(1):87–101. 10.1017/S0033291715001567. 26347066

[14] Yung AR Yuen HP McGorry PD Phillips LJ Kelly D Dell’Olio M Mapping the onset of psychosis: the comprehensive assessment of at-risk mental states. Aust N Z J Psychiatry (2005) 39(11–12):964–71. 10.1080/j.1440-1614.2005.01714.x 16343296

[15] McGlashan T Structured Interview for Prodromal Syndromes (SIPS).. New Haven, CT: Yale University (2001).

[16] Schultze-Lutter F Subjective symptoms of schizophrenia in research and the clinic: the basic symptom concept. Schizophr Bull (2009) 35(1):5–8. 10.1093/schbul/sbn139 19074497 PMC2643966

[17] Van Os J Delespaul P Toward a world consensus on prevention of schizophrenia. Dialogues Clin Neurosci(2005) 7(1):53–67.. 16060596 10.31887/DCNS.2005.7.1/jvanos PMC3181724

[18] Addington J Cornblatt BA Cadenhead KS Cannon TD McGlashan TH Perkins DO At clinical high risk for psychosis: outcome for nonconverters. Am J Psychiatry (2011) 168(8):800–5. 10.1176/appi.ajp.2011.10081191 PMC3150607 21498462

[19] Schultze-Lutter F Schimmelmann BG Ruhrmann S Michel C ‘A rose is a rose is a rose’, but at-risk criteria differ. Psychopathology (2013) 46(2):75–87. 10.1159/000339208 22906805

[20] Kuipers E Yesufu-Udechuku A Taylor C Kendall T Management of psychosis and schizophrenia in adults: summary of updated NICE guidance. BMJ (2014) 348:g1173. 10.1136/bmj.g1173 24523363

[21] Carpenter WT Anticipating DSM-V: should psychosis risk become a diagnostic class? Schizophr Bull (2009) 35(5):841–3. 10.1093/schbul/sbp071 PMC2728821 19633215

[22] Association AP Diagnostic and Statistical Manual of Mental Disorders. 5th ed Washington, DC: The Association (2013).

[23] Schultze-Lutter F Michel C Ruhrmann S Schimmelmann BG Prevalence and clinical significance of DSM-5-attenuated psychosis syndrome in adolescents and young adults in the general population: the Bern Epidemiological At-Risk (BEAR) study. Schizophr Bull (2014) 40(6):1499–508. 10.1093/schbul/sbt171 PMC4193691 24353096

[24] Carpenter WT van Os J Should attenuated psychosis syndrome be a DSM-5 diagnosis? Am J Psychiatry (2011) 168(5):460–3. 10.1176/appi.ajp.2011.10121816 21536700

[25] Davies C Cipriani A Ioannidis JPA Radua J Stahl D Provenzani U Lack of evidence to favor specific preventive interventions in psychosis: a network meta-analysis. World Psychiatry (2018) 17(2):196–209. 10.1002/wps.20526 29856551 PMC5980552

[26] Fusar-Poli P Borgwardt S Bechdolf A Addington J Riecher-Rossler A Schultze-Lutter F The psychosis high-risk state: a comprehensive state-of-the-art review. JAMA Psychiatry (2013) 70(1):107–20. 10.1001/jamapsychiatry.2013.269 PMC4356506 23165428

[27] Fusar-Poli P Nelson B Valmaggia L Yung AR McGuire PK Comorbid depressive and anxiety disorders in 509 individuals with an at-risk mental state: impact on psychopathology and transition to psychosis. Schizophr Bull (2014) 40(1):120–31. 10.1093/schbul/sbs136 PMC3885287 23180756

[28] Lin A Wood SJ Nelson B Beavan A McGorry P Yung AR Outcomes of nontransitioned cases in a sample at ultra-high risk for psychosis. Am J Psychiatry (2015) 172(3):249–58. 10.1176/appi.ajp.2014.13030418 25727537

[29] Brandizzi M Valmaggia L Byrne M Jones C Iwegbu N Badger S Predictors of functional outcome in individuals at high clinical risk for psychosis at six years follow-up. J Psychiatr Res (2015) 65:115–23. 10.1016/j.jpsychires.2015.03.005 25837413

[30] Devoe DJ Farris MS Townes P Addington J Attenuated psychotic symptom interventions in youth at risk of psychosis: a systematic review and meta-analysis. Early Intervention Psychiatry (2018) 13(1):3–17. 10.1111/eip.12677 PMC6230498 29749710

[31] Wampold BE Mondin GW Moody M Stich F Benson K Ahn HN A meta-analysis of outcome studies comparing bona fide psychotherapies: Empirically, “all must have prizes”. Psychol Bull (1997) 122(3):203–15. 10.1037//0033-2909.122.3.203

[32] Birchwood M Lester H McCarthy L Jones P Fowler D Amos T The UK national evaluation of the development and impact of Early Intervention Services (the National EDEN studies): study rationale, design and baseline characteristics. Early Intervention Psychiatry (2014) 8(1):59–67. 10.1111/eip.12007 23347742

[33] Faraone SV Asherson P Banaschewski T Biederman J Buitelaar JK Ramos-Quiroga JA Attention-deficit/hyperactivity disorder. Nat Rev Dis Primers (2015) 1:15020. 10.1038/nrdp.2015.20 27189265

[34] Carbon M Correll CU Clinical predictors of therapeutic response to antipsychotics in schizophrenia. Dialogues Clin Neurosci(2014) 16(4):505–24. 10.31887/DCNS.2014.16.4/mcarbon PMC4336920 25733955

[35] Devoe DJ Farris MS Townes P Addington J Interventions and social functioning in youth at risk of psychosis: a systematic review and meta-analysis. Early Intervention Psychiatry (2018) 13(2):169–80. 10.1111/eip.12689 29938910

[36] Debbané M Benmiloud J Salaminios G Solida-Tozzi A Armando M Fonagy P Mentalization-based treatment in clinical high-risk for psychosis: a rationale and clinical illustration. J Contemp Psychother (2016) 46(4):217–25. 10.1007/s10879-016-9337-4

[37] Debbané M Salaminios G Luyten P Badoud D Armando M Solida Tozzi A Attachment, neurobiology, and mentalizing along the psychosis continuum. Front Hum Neurosci (2016) 10:406. 10.3389/fnhum.2016.00406 27597820 PMC4992687

[38] Blakemore SJ Development of the social brain in adolescence. J R Soc Med (2012) 105(3):111–6. 10.1258/jrsm.2011.110221 PMC3308644 22434810

[39] Crone EA Ridderinkhof KR The developing brain: from theory to neuroimaging and back. Dev Cogn Neurosci (2011) 1(2):101–9. 10.1016/j.dcn.2010.12.001 PMC6987573 22436435

[40] Miller AB Lenzenweger MF Schizotypy, social cognition, and interpersonal sensitivity. Pers Disord (2012) 3(4):379–92. 10.1037/a0027955 22642463

[41] Gibson CM Penn DL Prinstein MJ Perkins DO Belger A Social skill and social cognition in adolescents at genetic risk for psychosis. Schizophr Res (2010) 122(1–3):179–84. 10.1016/j.schres.2010.04.018 PMC3132072 20570111

[42] Fonagy P Gergely G Jurist EL Target M Affect regulation, mentalization, and the development of the self. New York: Other Press (2002).

[43] Fonagy P Luyten P Allison E Epistemic petrification and the restoration of epistemic trust: a new conceptualization of borderline personality disorder and its psychosocial treatment. J Pers Disord (2015) 29(5):575–609. 10.1521/pedi.2015.29.5.575 26393477

[44] Fonagy P Steele M Steele H Higgitt A Target M The Emanuel Miller Memorial Lecture 1992. The theory and practice of resilience. J Child Psychol Psychiatry (1994) 35(2):231–57. 10.1111/j.1469-7610.1994.tb01160.x 8188797

[45] Dumontheil I Apperly IA Blakemore SJ Online usage of theory of mind continues to develop in late adolescence. Dev Sci (2010) 13(2):331–8. 10.1111/j.1467-7687.2009.00888.x 20136929

[46] Hillebrandt H Dumontheil I Blakemore SJ Roiser JP Dynamic causal modelling of effective connectivity during perspective taking in a communicative task. Neuroimage (2013) 76:116–24. 10.1016/j.neuroimage.2013.02.072 23507383

[47] van Donkersgoed RJ Wunderink L Nieboer R Aleman A Pijnenborg GH Social cognition in individuals at ultra-high risk for psychosis: a meta-analysis. PLoS One (2015) 10(10):e0141075. 10.1371/journal.pone.0141075 26510175 PMC4624797

[48] Addington J Penn D Woods SW Addington D Perkins DO Social functioning in individuals at clinical high risk for psychosis. Schizophr Res (2008) 99(1–3):119–24. 10.1016/j.schres.2007.10.001 PMC2292799 18023329

[49] Bora E Murray RM Meta-analysis of cognitive deficits in ultra-high risk to psychosis and first-episode psychosis: do the cognitive deficits progress over, or after, the onset of psychosis? Schizophr Bull (2014) 40(4):744–55. 10.1093/schbul/sbt085 PMC4059428 23770934

[50] Jang JH Shin NY Shim G Park HY Kim E Jang GE Longitudinal patterns of social functioning and conversion to psychosis in subjects at ultra-high risk. Aust N Z J Psychiatry (2011) 45(9):763–70. 10.3109/00048674.2011.595684 21827349

[51] Cannon TD Chung Y He G Sun D Jacobson A van Erp TG Progressive reduction in cortical thickness as psychosis develops: a multisite longitudinal neuroimaging study of youth at elevated clinical risk. Biol Psychiatry (2015) 77(2):147–57. 10.1016/j.biopsych.2014.05.023 PMC4264996 25034946

[52] Frith CD Frith U The neural basis of mentalizing. Neuron (2006) 50(4):531–4. 10.1016/j.neuron.2006.05.001 16701204

[53] Debbané M Van der Linden M Balanzin D Billieux J Eliez S Associations among metacognitive beliefs, anxiety and positive schizotypy during adolescence. J Nerv Ment Dis (2012) 200(7):620–6. 10.1097/NMD.0b013e31825bfc1a 22759941

[54] Debbané M Van der Linden M Gex-Fabry M Eliez S Cognitive and emotional associations to positive schizotypy during adolescence. J Child Psychol Psychiatry (2009) 50(3):326–34. 10.1111/j.1469-7610.2008.01961.x 19175821

[55] Kim HS Shin NY Jang JH Kim E Shim G Park HY Social cognition and neurocognition as predictors of conversion to psychosis in individuals at ultra-high risk. Schizophr Res (2011) 130(1–3):170–5. 10.1016/j.schres.2011.04.023 21620681

[56] Piskulic D Liu L Cadenhead KS Cannon TD Cornblatt BA McGlashan TH Social cognition over time in individuals at clinical high risk for psychosis: findings from the NAPLS-2 cohort. Schizophr Res (2016) 171(1–3):176–81. 10.1016/j.schres.2016.01.017 PMC5037438 26785807

[57] Davidson CA Piskulic D Addington J Cadenhead KS Cannon TD Cornblatt BA Age-related trajectories of social cognition in youth at clinical high risk for psychosis: an exploratory study. Schizophr Res (2018) 201:130–6. 10.1016/j.schres.2018.05.001 PMC8130825 29751984

[58] Meehl PE Schizotaxia, schizotypy, schizophrenia. Am Psychol (1962) 17:827–38. 10.1037/h0041029

[59] Meehl PE Toward an integrated theory of schizotaxia, schizotypy, and schizophrenia. J Pers Disord (1990) 4:1–99. 10.1521/pedi.1990.4.1.1

[60] Debbané M Eliez S Badoud D Conus P Fluckiger R Schultze-Lutter F Developing psychosis and its risk States through the lens of schizotypy. Schizophr Bull (2015) 41(Suppl 2):S396–407. 10.1093/schbul/sbu176 PMC4373628 25548386

[61] Bartels-Velthuis AA Blijd-Hoogewys EM van Os J Better theory-of-mind skills in children hearing voices mitigate the risk of secondary delusion formation. Acta Psychiatr Scand (2011) 124(3):193–7. 10.1111/j.1600-0447.2011.01699.x 21426312

[62] Peters E Ward T Jackson M Morgan C Charalambides M McGuire P Clinical, socio-demographic and psychological characteristics in individuals with persistent psychotic experiences with and without a “ need for care ”. World Psychiatry: Official Journal of the World Psychiatric Association (WPA). (2016) 15(1):41–52). 10.1002/wps.20301 26833608 PMC4780307

[63] Chadwick P Hember M Symes J Peters E Kuipers E Dagnan D Responding mindfully to unpleasant thoughts and images: Reliability and validity of the Southampton mindfulness questionnaire (SMQ). Br J Clin Psychol (2008) 47:451–5. 10.1348/014466508X314891 18573227

[64] Dudley J Eames C Mulligan J Fisher N Mindfulness of voices, self-compassion, and secure attachment in relation to the experience of hearing voices. Br J Clin Psychol (2018) 57(1):1–17. 10.1111/bjc.12153 28801978 PMC5811822

[65] Choi-Kain LW Gunderson JG Mentalization: ontogeny, assessment, and application in the treatment of borderline personality disorder. Am J Psychiatry (2008) 165(9):1127–35. 10.1176/appi.ajp.2008.07081360 18676591

[66] Fonagy P Luyten P A developmental, mentalization-based approach to the understanding and treatment of borderline personality disorder. Dev Psychopathol (2009) 21(4):1355–81. 10.1017/S0954579409990198 19825272

[67] Bevington D Fuggle P Fonagy P Applying attachment theory to effective practice with hard-to-reach youth: the AMBIT approach. Attachment & Human Development (2015) 17(2):157–74. 10.1080/14616734.2015.1006385 25782529

[68] Rossouw TI Fonagy P Mentalization-based treatment for self-harm in adolescents: a randomized controlled trial. J Am Acad Child Adolesc Psychiatry (2012) 51(12):1304–13 e3. 10.1016/j.jaac.2012.09.018 23200287

[69] Weijers J Ten Kate C Eurelings-Bontekoe E Viechtbauer W Rampaart R Bateman A Mentalization-based treatment for psychotic disorder: protocol of a randomized controlled trial. BMC Psychiatry (2016) 16:191. 10.1186/s12888-016-0902-x 27278250 PMC4898403

[70] Lana F Marcos S Mollà L Vilar A Pérez V Romero M Mentalization based group psychotherapy for psychosis: a pilot study to assess safety, acceptance and subjective efficacy. Int J Psychol Psychoanal (2015) 1(1):1–6. 10.23937/2572-4037.1510007

[71] Bateman AW Fonagy P Randomized controlled trial of outpatient mentalization-based treatment versus structured clinical management for borderline personality disorder. Am J Psychiatry (2009) 166(12):1355–64. 10.1176/appi.ajp.2009.09040539 19833787

[72] Bateman AW Fonagy P 8-year follow-up of patients treated for borderline personality disorder: mentalization-based treatment versus treatment as usual. Am J Psychiatry (2008) 165(5):631–8. 10.1176/appi.ajp.2007.07040636 18347003

[73] Bateman AW Fonagy P Effectiveness of partial hospitalization in the treatment of borderline personality disorder: a randomized controlled trial. Am J Psychiatry (1999) 156(10):1563–9. 10.1176/ajp.156.10.1563 10518167

[74] Bateman AW Fonagy P Handbook of mentalizing in mental health practice. Arlington: American Psychiatric Publishing (2012).

[75] Brent BK Holt DJ Keshavan M Seidman L Fonagy P Mentalization-based treatment for psychosis: linking an attachment-based model to the psychotherapy for impaired mental state understanding in people with psychotic disorders. Isr J Psychiatry Relat Sci (2014) 51(1):17–24. 24858631

[76] Brent BK A mentalization-based approach to the development of the therapeutic alliance in the treatment of schizophrenia. J Clin Psychol (2015) 71(2):146–56. 10.1002/jclp.22150 25557537

[77] Brent B Fonagy P A mentalization-based treatment approach to disturbances of social understanding in schizophrenia. In: Lysaker PH Dimaggio G Brüne M, editors. Social cognition and metacognition in schizophrenia. San Diego: Elsevier (2014). p. 245–57. 10.1016/B978-0-12-405172-0.00015-6

[78] McGorry PD Purcell R Hickie IB Yung AR Pantelis C Jackson HJ Clinical staging: a heuristic model for psychiatry and youth mental health. Med J Aust(2007) 187(7 Suppl):S40–2. 10.5694/j.1326-5377.2007.tb01335.x 17908024

[79] Nelson B Amminger GP Yuen HP Wallis N JK M Dixon L Staged treatment in early psychosis: a sequential multiple assignment randomised trial of interventions for ultra high risk of psychosis patients. Early Intervention Psychiatry (2018) 12(3):292–306. 10.1111/eip.12459 PMC6054879 28719151

[80] Millan MJ Andrieux A Bartzokis G Cadenhead K Dazzan P Fusar-Poli P Altering the course of schizophrenia: progress and perspectives. Nat Rev Drug Discov (2016) 15(7):485–515. 10.1038/nrd.2016.28 26939910

[81] Rapado-Castro M McGorry PD Yung A Calvo A Nelson B Sources of clinical distress in young people at ultra high risk of psychosis. Schizophr Res (2015) 165(1):15–21. 10.1016/j.schres.2015.03.022 25890793

[82] Hosman C Jane-Llopis E Saxena S Prevention of mental disorders: effective interventions and policy options. Oxford: Oxford University Press (2005).

[83] Twemlow SW Fonagy P Sacco FC A developmental approach to mentalizing communities: II. The Peaceful Schools experiment. Bull Menninger Clin (2005) 69(4):282–304. 10.1521/bumc.2005.69.4.282 16370790

[84] Twemlow SW Fonagy P Sacco FC A developmental approach to mentalizing communities: I. A model for social change. Bull Menninger Clin (2005) 69(4):265–81. 10.1521/bumc.2005.69.4.265 16370789

[85] Bak PL Midgley N Zhu JL Wistoft K Obel C The Resilience Program: preliminary evaluation of a mentalization-based education program. Front Psychol (2015) 6:1–6. 10.3389/fpsyg.2015.00753 26136695 PMC4468359

[86] Asen E Fonagy P Mentalizing family violence part 2: techniques and interventions. Fam Process (2017) 56(1):22–44. 10.1111/famp.12276 28133724

[87] Asen E Fonagy P Mentalizing family violence part 1: conceptual framework. Fam Process (2017) 56(1):6–21. 10.1111/famp.12261 27861799

[88] Fuggle P Bevington D Cracknell L Hanley J Hare S Lincoln J The Adolescent Mentalization-based Integrative Treatment (AMBIT) approach to outcome evaluation and manualization: adopting a learning organization approach. Clin Child Psychol Psychiatry (2015) 20(3):419–35. 10.1177/1359104514521640 24595808

[89] Debbané M Bateman A Psychosis. In: Bateman A Fonagy P, editors. handbook of mentalizing in mental health practice. 2nd ed Washington: American Psychiatric Association Publishing (2019). p. 443–58.

[90] Bateman A Fonagy P Handbook of mentalizing in mental health practice, 2nd ed Washington: American Psychiatric Association Publishing (2019).

[91] Weijers J Fonagy P Eurelings-Bontekoe E Termorshuizen F Viechtbauer W Selten JP Mentalizing impairment as a mediator between reported childhood abuse and outcome in nonaffective psychotic disorder. Psychiatry Res (2018) 259:463–9. 10.1016/j.psychres.2017.11.010 29145104

